# Reduction of Glycolysis Intermediate Concentrations in the Cerebrospinal Fluid of Alzheimer’s Disease Patients

**DOI:** 10.3389/fnins.2019.00871

**Published:** 2019-08-21

**Authors:** Nick Bergau, Stephan Maul, Dan Rujescu, Andreas Simm, Alexander Navarrete Santos

**Affiliations:** ^1^Department of Cell and Metabolic Biology, Leibniz Institute of Plant Biochemistry, Halle (Saale), Germany; ^2^Department of Psychiatry, Psychotherapy and Psychosomatics, Martin-Luther-University Halle-Wittenberg, Halle (Saale), Germany; ^3^Center for Basic Medical Research, Martin-Luther-University Halle-Wittenberg, Halle (Saale), Germany; ^4^Clinic for Cardiac Surgery, University Hospital, Martin-Luther-University Halle-Wittenberg, Halle (Saale), Germany

**Keywords:** Alzheimer’s disease, metabolomics, glycolysis intermediates, sugar phosphates, cerebrospinal fluid, LC-MS/MS

## Abstract

The profile of 122 metabolites in the cerebrospinal fluid (CSF) of patients suffering from Alzheimer’s disease (AD) and controls was studied. Among the 122 metabolites analyzed, 61 could be detected. Statistically significant differences between the AD and control group were only detected for metabolites of the glycolysis. Thus, accurate quantification of 11 glycolytic metabolites was done. We detected a significant reduction of five of them, namely phosphoenolpyruvate, 2-phosphoglycerate, 3-phosphoglycerate, pyruvate and dihydroxyacetone phosphate in the AD CSF compared to controls. These results correlate with the known reduction of glucose metabolism in the brain of patients with AD and indicate that metabolic analysis of the central carbon metabolism can be a potential tool in AD diagnostic. Although the Receiver operating characteristic (ROC) analyses of the metabolites do not reach the level of the diagnostic informativity of AD biomarkers, the combination of specific glycolysis metabolites with the established biomarkers may lead to an improvement in sensitivity and specificity.

## Introduction

Alzheimer’s disease (AD) is a progressive neurodegenerative disorder that leads to cognitive decline, impairment of activities of daily living and neuropsychological abnormalities. Typical hallmarks of AD in the brain are extracellular deposits of amyloid β (Aβ), a cleavage product of amyloid precursor protein (APP), and neuronal deposits of neurofibrillary tangles (NFTs) consisting of hyperphosphorylated Tau protein (Bird, 1993–2019). Various imaging and cerebrospinal fluid (CSF) biomarkers are helpful for diagnosing AD and distinguish AD from other forms of dementia (Jack et al., 2018). However, diagnosis can be challenging, particularly in the prodromal and early stages of AD, and research into biomarkers that improve the diagnostic process and provide further insights into the underlying causes and mechanisms is therefore important.

In this context, metabolomics opens up new perspectives for understanding the mechanism behind dementia and in particular AD. Metabolomic studies conducted in both animal models and AD patients have been summarized in recent reviews (Trushina and Mielke, 2014; Wilkins and Trushina, 2018). In AD patients, these studies identified key-disease-related metabolic changes and disease-progression-related changes (Toledo et al., 2017). Six metabolic pathways of the central metabolism, as well as glycerophospholipid metabolism, were found predominantly altered in AD brains (Paglia et al., 2016). In particular, amino acid profiling among patients with AD and healthy controls have been conducted summarized in Socha et al. (2019). At this, significant changes in the amino acid concentrations were observed in AD patients compared to healthy controls, which in combination with other studies (Enche Ady et al., 2017; Wilkins and Trushina, 2018; Grasso, 2019) points to a high value of metabolic profiling as a method for the diagnosis of AD. However, the knowledge gained so far is not sufficient to use metabolomics for differential diagnostic considerations.

The well characterized CSF biomarker profile (decrease of amyloid β 1-42 (Aβ1-42) associated with increase of both total Tau (t-Tau) and phosphorylated Tau (p-Tau)) is a helpful tool for AD diagnosis. This profile reflects key aspects of disease pathogenesis, such as neuronal and axonal degeneration, phosphorylation of tau with tangle formation, and oligomerization, aggregation and deposition of the Aβ1-42 peptide into plaques (Blennow and Hampel, 2003). To which extension these analytes correlate with metabolites in the CSF of AD patients has not been exhaustively examined.

In this study, AD and non-AD CSF samples of patients characterized according to clinical and imaging data as well as biomarker profiles were analyzed by MS. The aim of this work was to identify metabolites and pathways in the CSF of AD patients which reflect the disease and correlate with the biochemical biomarkers.

## Materials and Methods

### Collection and Analysis of Cerebrospinal Fluid and Criteria for AD Diagnosis

Patients were recruited at the Department of Psychiatry, Psychotherapy and Psychosomatics of the University Hospital of the Martin-Luther-University Halle-Wittenberg. CSF samples were collected from patients who were examined for memory disorder. Samples of 22 AD patients with a mean age of 77.1 ± 6.6 years and 33 controls with a mean age of 68.8 ± 9.1 years (Table 1) were analyzed (*p* = 0.001). AD diagnosis was determined by clinical findings according to NIA-AA guidelines (McKhann et al., 2011), under additional consideration of CSF results (Abeta 1-40, Abeta 1-42, total-Tau and p-Tau; Table 1), cognitive tests, CT/MRI and FDG-PET. CSF samples from participants who had no indication of AD were assigned to the non-AD group. The cognitive impairment in the non-AD group was caused by other disorders. In addition to patients with MCI in whom there was no evidence of AD in CSF, CT/MRI and FDG-PET (Ten subjects), this group included patients with cognitive impairments due to pre-existing schizophrenia or schizoaffective disorder (Seven subjects), depression (Four subjects), personality disorder (One subject), alcohol-toxic brain damage (Three subjects), organic psychoses (Three subjects) and subjective cognitive decline (Five subjects). Concentrations of Abeta 1-40, Abeta 1-42, p-Tau (181P) and total-Tau in CSF were determined by using the appropriate ELISA-kits (INNOTEST^®^ β-AMYLOID_(__1__–__40__)_, INNOTEST^®^ β-AMYLOID_(__1__–__42__)_, INNOTEST^®^ PHOSPHO-TAU_(__181__P)_, INNOTEST^®^ hTAU-Ag; Fujirebio, Ghent, Belgium) as part of the clinical routine. As comorbidity, type 2 diabetes mellitus was present in 3 AD patients and ten patients of the non-AD group, while none of the AD patients and 2 non-AD controls reported a history of stroke.

**TABLE 1:** Value of the biochemical biomarkers (mean ± standard deviation) used for discrimination between AD and non-AD patients.

	**Control**	**AD**	***p*-value**
N	33 (47.2% Male)	22 (22.7% Male)	
Aβ1-40 (pg/ml)	10863.7 ± 4014.6	15413.9 ± 8628.6	5.2 × 10^–3^
Aβ1-42 (pg/ml)	943.4 ± 287.6	465.7 ± 137.9	<1.0 × 10^–4^
p-Tau (pg/ml)	51.2 ± 30.9	93.7 ± 43.2	2.9 × 10^–3^
Total-Tau (pg/ml)	282.3 ± 194.6	717.4 ± 507.9	3.0 × 10^–4^
Aβ1-42^∗^10/Aβ1-40	0.9 ± 0.2	0.39 ± 0.2	<1.0 × 10^–4^

*Least squares multiple regression analysis was used to analyze the relationship between the dependent variables (biomarkers) and the independent variables (age, gender, disease). A *p* < 0.05 was considered statistically significant.*

The collected CSF samples were aliquoted and stored at −80°C. Samples stained due to blood contamination were eliminated from further analysis. The study was performed according to the Declaration of Helsinki. The data collection and analysis were approved by the ethical committee of the Martin-Luther-University Halle-Wittenberg (medical faculty).

### LC-MS Analysis

Prior to LC-MS analysis liquor samples were filtrated (PVDF 96 well Filter 0.2 μm, Corning). Analysis of glycolysis intermediates was performed on an Acquity UPLC (Waters, Eschborn, Germany) coupled to a QTrap 6500 mass analyzer (SCIEX, Toronto, Canada). Thereby the separation of the metabolites was done by ion-pair reversed phase chromatography on a Nucleoshell RP18 plus column (2.7 mm, 2 mm × 150 mm, Macherey Nagel, Düren, Germany) using a gradient of 10 mM tributylamine (solvent A) and acetonitrile (solvent B) with the following gradient: 0–2 min isocratic 98% A, 2–18 min linear from 98% to 64% A, 18–21 min linear from 64 to 5% A, 21–22.5 min isocratic 5% A, 22.5–22.52 min linear from 5 to 98% A, 22.52–24 min isocratic 98% A. The flow rate was set to 400 μl/min. The injection volume was 10 μl and the column temperature was set to 40°C. MS detection was done by ESI-MS in negative mode with the following parameters: curtain gas = 40 psi, ionspray voltage = −4500 V, Entrance Potential = −10, temperature = 450°C, gas1 = 60 psi, gas2 = 70 psi. The detection of the metabolites was done by multiple reaction monitoring (see Supplementary Table S1) and the identity of them was confirmed by authentic standards (all from Sigma-Aldrich, Taufkirchen, Germany). The quantification of the glycolysis intermediates was done based on a serial dilution of authentic standards in the range from 3 to 12500 μg/l [pyruvic acid (PYR), phosphoenolpyruvate (PEP), 2-phosphoglycerate (2PG), glucose-6-phosphate (G6P) and dihydroxyacetone phosphate (DHAP)] in water (for typical standard curves see Supplementary Figure S1). For glucose the concentrations ranged from 312.5 to 2500 mg/l. The quantification of 3PG was based on the dilution series of its isomer 2PG. It has to be noted that the peaks of the isomers glucose-6-phosphate (G6P) and fructose-6-phosphate (F6P) could not be separated clearly and therefore were integrated as the sum of both analytes. The intraday and interday reproducibility of the used method was analyzed by measuring standard solutions and CSF samples (*n* = 3) 3 times on the same day as well as 24 h later. At this the intraday reproducibility for the standard solutions of the glycolysis intermediates ranged from 2.1 to 9.2% while interday reproducibility ranged from 1.6 to 15.5%. For the CSF samples the intraday reproducibility ranged from 2.1 to 10.8% while interday reproducibility ranged from 1.2 to 18.8%. The LOQs of the quantified metabolites were 0.11 μM (2PG + 3PG); 0.53 μM (PYR); 0.09 μM (PEP); 0.02 μM (DHAP); 0.01 μM (G6P + F6P); 0.13 μM (Glucose) and at this markedly lower than the concentrations of the detected metabolites.

### Statistical Analysis

Statistical analyses were performed by using the MedCalc software (version 19.0.3). Least squares multiple regression analysis was used to analyze the relationship between the dependent variables (metabolites, biomarkers) and the independent variables (age, gender, disease). ROC curve analyses were also performed. The Delong’s test was used for comparison of the different ROC curves. A *P*-value less than 0.05 was considered statistically significant.

## Results

As shown in Tables 1, 3, the analyzed patient collective can clearly be distinguished into the non-AD and AD groups. The cut-off values used for the distinction between AD and non-AD were determined by using the ROC curves of the biomarkers. The cut-off values were obtained for the best-achieved sensitivity and specificity. The AD patients showed decreased Aβ1-42 levels and increased Aβ1-40 levels. Correspondently, Aβ ratio was significantly decreased in the AD group. Both t-Tau and p-Tau were significantly increased in AD patients. This is in line with the well-known and characteristic changes as summarized in a recent review (Blennow and Zetterberg, 2018). Additionally, cognitive performance measured by Mini-Mental Status Examination (MMSE) revealed significant differences with a mean score of 19.8 ± 6.4 for AD patients and 26 ± 3.9 for non-AD patients (*p* = 0.0001).

It is important to remark that not all of the AD and non-AD patients fulfilled the cut-off criteria for Aβ1-42, p-Tau and t-Tau. In the AD group, 2 patients did not meet the Aβ1-42 threshold and 4 patients did not meet the t-Tau and the p-Tau cut-off. In the control group, 4 participants did not fulfill the cut-off criteria for Aβ1-42 and p-Tau and 3 participants did not meet the threshold for t-Tau. The reason for this was that the grouping was not exclusively based on the biomarker profiles, but also on the clinical and imaging data. This explains why the ROC curves for the different biomarkers do not show 100% specificity and sensitivity.

### Detection of Metabolites in the CSF

From the 122 metabolites analyzed in our study, 61 could be detected in all AD (*n* = 22) and control subjects (*n* = 33; Supplementary Table S1). Among the 61 metabolites detectable in all subjects, only the metabolites related to the glucose metabolism showed significant differences between the AD and non-AD groups. The summarized statistical analysis of the metabolites detected in the CSF is shown in Supplementary Table S2. In order to evaluate these differences more precisely, 11 metabolites of the glycolysis (see Table 2) were quantified by their authentic standards.

**TABLE 2:** Concentrations of glycolysis intermediates (mean ± standard deviation) in the AD and in the non-AD groups.

**Metabolite (μM)**	**Control**	**AD**	***p*-value**
**DHAP**	0.09 ± 0.03	0.07 ± 0.02	**3.8 × 10^–3^**
Glucose	2815.89 ± 1350.10	2168.55 ± 915.83	6.6 × 10^–2^
G6P + F6P	0.23 ± 0.04	0.22 ± 0.05	3.8 × 10^–1^
**2PG**	0.22 ± 0.02	0.21 ± 0.01	**1.3 × 10^–2^**
**3PG**	0.62 ± 0.11	0.50 ± 0.10	**1.0 × 10^–4^**
**PEP**	0.50 ± 0.09	0.40 ± 0.07	**<1.0 × 10^–4^**
**PYR**	82.10 ± 18.96	72.52 ± 19.63	**5.2 × 10^–3^**
BPG	*nd*	*nd*	
F-1,6-BP	*nd*	*nd*	
GAP	*nd*	*nd*	

*Least squares multiple regression analysis was used to analyze the relationship between the dependent variables (metabolites) and the independent variables (age, gender, disease). A *p* < 0.05 (bolded values) was considered statistically significant. G6P, glucose-6-phosphate; F6P, fructose-6-phosphate; DHAP, dihydroxyacetone phosphate; BPG, 2,3-bisphosphoglycerate; 2PG, 2-phosphoglycerate; 3PG, 3-phosphoglycerate; PEP, phosphoenolpyruvate; PYR, pyruvic acid; F-1,6-BP, fructose-1-6-biphosphate; GAP, glyceraldehyde-3-phosphate; nd, not detected.*

### Reduced Contents of Glycolysis Intermediates in AD Patients

**FIGURE 1:**
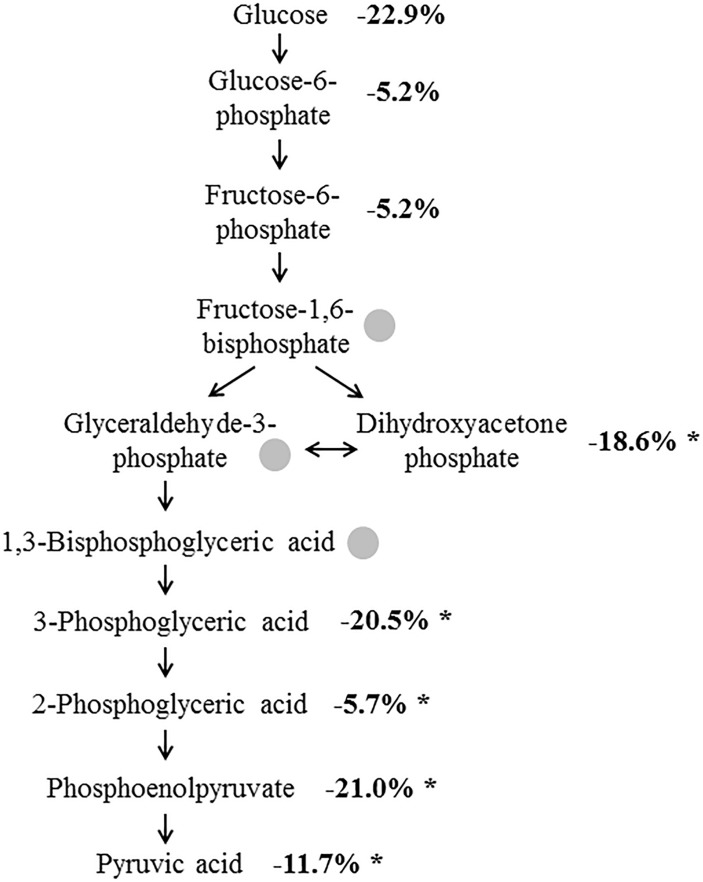
Altered concentration of glycolysis intermediates in AD. Numbers indicate the concentrations of the metabolites in the AD samples in percent compared to control samples. Intermediates marked by a gray dot could not be detected. ^∗^*p* ≤ 0.05.

The analysis of the glycolysis intermediates in the CSF of the 22 AD patients and the 33 non-AD patients revealed the presence of 8 out of the 11 analyzed compounds (see Table 2) in all samples. The concentrations of fructose 1-6-biphosphate, 2,3-bisphosphoglycerate and glyceraldehyde-3-phosphate were below the detection limit. All of the detectable compounds showed reduced concentrations in the AD samples compared to the non-AD group. Five of these eight metabolites were significantly reduced, with the highest changes and lowest *p*-values found for PEP (21% reduction, *p* < 0.0001), 3PG (20.5% reduction, *p* = 0.0001) and DHAP (18.6% reduction, *p* = 0.0038; Figure 1), indicating reduced glycolysis activity reflected in CSF of AD patients. A detailed Supplementary Table S3 shows the summary of the statistical analysis for the metabolites of the glycolysis.

Except for PYR, all the metabolites showed a significant positive correlation with Aβ1-42 and Aβ1-42/Aβ1-40. The highest correlation was obtained when comparing between Aβ1-42/Aβ1-40 and 3PG (*r* = 0.49; *p* = 0.0001); Supplementary Figure S2. The metabolites of the glycolysis showed a negative correlation with t-Tau and p-Tau without statistical significance.

### ROC Curve Analysis

In order to evaluate the power of glycolysis metabolites as diagnostic instrument, ROC curve analyses for the metabolites showing significant changes between the AD and non-AD groups were done. The results are summarized in Table 3. Regarding the metabolites of the glycolysis, only those with significant changes were analyzed. The best performance was achieved by PEP with a sensitivity of 63.6% and a specificity of 90.9%. In order to check for the best performance, we then tested different combination of the metabolites. Combinations other than those shown in the Table 3 have not performed better and are not shown. With the combination PEP + DHPA and PEP + 2PG we observed a slight increase in sensitivity (72.7%) and a slight decrease in specificity (81.8%) compared to single metabolites. These results do not reach the performance of the classical biomarkers Aβ1-42, p-Tau and t-Tau (Table 3). In the next step, AD biomarkers were combined with the glycolysis metabolites. Table 3 shows the best performances achieved with different combinations. We defined one combination as “amyloid normalization by summation” (ANS).

ANS = (Aβ1-42/Aβ1-40) × 5 + (PEP + DHAP + 2PG + 3PG)

**TABLE 3:** ROC curve analyses and cut-off values of AD biomarkers and metabolites of the glycolysis.

**Metabolites**	**Cut-off**	**AUC**	**Sensitivity (%)**	**Specificity (%)**
PYR (pg/ml)	≤ 68.48	0.67	59.10	78.80
PEP (pg/ml)	≤ 0.41	0.83	**63.60**	**90.90**
DHAP (pg/ml)	≤ 0.06	0.68	45.50	90.90
2PG (pg/ml)	≤ 0.22	0.73	77.30	63.60
3PG (pg/ml)	≤ 0.61	0.80	95.50	54.50
**Metabolites summation**				
PEP + DHAP	≤ 0.52	0.80	**72.70**	**81.80**
PEP + DHAP + 2PG	≤ 0.73	0.79	68.20	81.80
PEP + DHAP + 2PG + 3PG	≤ 1.32	0.80	68.20	81.80
PEP + 2PG	≤ 0.64	0.82	**72.70**	**81.80**
PEP + 3PG	≤ 0.98	0.81	72.70	78.80
DHAP + 2PG	≤ 0.33	0.68	95.50	42.40
2PG + 3PG	≤ 0.82	0.80	95.50	53.10
**Biomarkers**				
Aβ1-40 (pg/ml)	> 14265.00	0.65	54.50	78.10
Aβ1-42 (pg/ml)	≤ 589.00	0.92	**87.50**	**90.90**
Aβ1-42/Aβ1-40	≤ 0.06	0.93	**90.90**	**90.60**
p-Tau (pg/ml)	> 65.30	0.83	**81.00**	**83.90**
t-T (pg/ml)	> 406.00	0.85	**81.80**	**87.50**
**Biomarkers and Metabolites**				
ANS	≤ 1.59	0.94	**95.50**	**93.90**
p-Tau/PEP	> 149.61	0.87	**90.50**	**93.50**
p-Tau/(PEP + DHAP)	> 121.63	0.87	**90.50**	**90.30**
t-Tau/PEP	> 952.63	0.87	**81.80**	**96.90**
t-Tau/(PEP + DHAP)	> 794.55	0.88	**81.80**	**93.70**

*ANS, amyloid normalization by summation. ANS = (Aβ1-42/Aβ1-40) × 5 + (PEP + DHAP + 2PG + 3PG). The bolded values denote the best performance (sensitivity, specificity) achieved.*

This combination performed better than Aβ1-42/Aβ1-40 alone with increased sensitivity (95.5%) and specificity (93.7%). In the equation, the factor 5 was empirically determined and was superior to the factor 10 normally used for the Aβ ratio.

We further observed an improvement in the performance of p-Tau and t-Tau in combination with the metabolites. The best performance achieved was by using the ratio p-Tau/PEP. All of the other metabolite combinations with p-Tau and t-Tau performed better than the biomarkers alone (Table 3). Comparisons of the ROC curves by using the Delong’s test showed no significant difference for ANS versus Aβ1-42/Aβ1-40 but for ANS versus p-Tau (*p* = 0.05), for p-Tau/PEP versus p-Tau (*p* = 0.01) and for p-Tau/(PEP + DHAP) versus p-Tau (*p* = 0.01).

## Discussion

In this study, we report on the analysis of 122 metabolites in the CSF of AD and non-AD patients by LC-MS. The classification into AD and non-AD was based on clinical and imaging data as well as CSF biomarkers, which can be used to classify cognitive impairment at an early stage (Falgàs et al., 2019).

Among the 122 metabolites presently analyzed, 61 were detected in the CSF samples independently of diagnosis. From the detected metabolites, only intermediates of the glycolysis were significantly altered in the CSF of AD versus non-AD samples. The metabolites of the glycolysis were decreased in the AD group and prompted us to analyze 11 of them in more detail. The further quantified metabolites were Glucose, G6P, F6P, DHAP, BPG, 2PG, 3PG, PEP, PYR, F-1,6-BP, and GAP.

Of the 11 analytes measured, PEP, 2PG, 3PG, PYR, and DHAP showed significant differences between the two groups. To our knowledge, this is the first report on the measurement of these metabolites in CSF in one and the same study. In an earlier study by Redjems-Bennani et al. (1998), among others, the concentrations of glucose, lactate, and pyruvate were measured in the CSF of AD and controls. In accordance with our study (AD 2.1 ± 0.9; controls 2.8 ± 1.3 mM), no significant difference regarding the glucose concentration was found (AD 2.6–4.7; controls 2.7–5.5 mM). In a recently published meta-analysis of CSF markers, Manyevitch et al. (2018) emphasized that there is no glucose deficit in the brain of AD patients as the glucose concentration was the same as in control brains. However, a significant decrease in pyruvate was observed, which is in line with our results. Pyruvate can be converted into lactate or into acetyl-CoA under oxidative conditions. Regarding the lactate concentration, we did not detect any differences between AD and non-AD; therefore lactate was not further analyzed. A statement about the conversion of pyruvate to acetyl-CoA cannot be done as acetyl-CoA was not detected in any of the CSF samples analyzed.

Perturbed cerebral glucose metabolism may be a critical contributor to the AD pathogenesis (Enche Ady et al., 2017). Our results support the observation that AD is associated with reduced glucose metabolism in the central nervous system as previously detected in cerebral imaging using MRI and FDG-PET (Verclytte et al., 2016). Furthermore, increased levels of proteins involved in the regulation of glucose metabolism in AD brains including glyceraldehyde-3-phosphate dehydrogenase, an enzyme involved in the catabolism of GAP to BPG was observed (Musunuri et al., 2014) and further supports, together with our results, aberrant glucose metabolism in AD patients. Moreover, under conditions of oxidative stress, the activities of key enzymes along the glycolytic pathway in AD are reduced and glycolysis is inhibited (Mamelak, 2012). The reduced levels of glycolytic metabolites in the CSF of AD patients found in this study may be partly explained by both of these mechanisms.

The AD-specific senile plaques in AD brains are co-localized with advanced glycation end products (AGE), and plaque-enriched fractions contain 3-fold higher AGE products than preparations of the age-matched non-demented controls (Lueth et al., 2005). In an early study by Gil et al., it was shown *in vitro* that the glycation rates of human hemoglobin increased with an increase of 2PG and 3PG concentrations (Gil et al., 2002). The relation between the decreased concentrations of 2PG and 3PG in the CSF of AD patients, as found in the present study, and the products of the glycation reaction must be further elucidated.

We are aware that the number of samples analyzed in this study is small and thus the results should be interpreted with caution. In addition, the groups differ in age as well. In this study, only patients with cognitive complaints were examined, with significant differences between the AD group and other causes of cognitive complaints. In order to be able to classify the differences even better, a comparison with healthy controls would be meaningful.

However, as the difference of 3PG and PEP between AD and non-AD patients is highly significant, we want to point to the applicability of metabolome analysis in the diagnostic process of neurodegenerative diseases. Indeed, in AD, robust and informative biomarkers detectable in the prodromal phase of the disease are still missing. A reduction in cerebral metabolism for glucose is a very early event in AD and the progressive reduction of the metabolic glucose rate correlates with the clinical disability (Mosconi et al., 2008). Therefore, it would be of interest to analyze metabolites of the glycolysis in CSF of patients with MCI or in a pre-symptomatic phase prior to conversion to AD, even if the focus here should be on the detection of blood biomarkers, since blood can be collected less invasively than CSF. Accordingly, it could be possible to elucidate if the reduced glycolysis activity in the brain of AD patients is a cause or an effect. The use of metabolomic markers in addition to the established biomarkers may improve diagnostic specificity and sensitivity, as our ROC curve analyses show. However, it is important to mention that although higher sensitivity and specificity were obtained by using the metabolites in combination with the biomarkers, significant differences were observed only by combining the metabolites with p-Tau and t-Tau, but not with Aβ1-42 or the ratio Aβ1-42/Aβ1-40. These results underline the high value of Aβ1-42 as a biomarker of AD. Further studies are necessary for supporting or disapproving the use of the metabolite and biomarker combinations described herein. Metabolomics could contribute to characterize the progression of AD as well as different disease stages. However, further investigations are also necessary for this purpose. Of particular interest is, on the one hand, how the metabolic alterations change in the course of the disease and, on the other hand, whether these changes also show up in other neurodegenerative diseases or whether this is an AD-specific effect. This will determine whether these glycolysis intermediates may be suitable for use in the diagnosis of AD.

In conclusion, we found a high correlation between the established CSF biomarkers Aβ1-42; Aβ1-42/Aβ1-40 and the metabolites PEP, 2PG, 3PG, and DHAP. However, it should be mentioned, that general conclusions on the diagnostic benefit of metabolic biomarkers are limited, as there are limitations of the present study regarding the small sample size, age differences between the AD- and non-AD groups and the inhomogeneous control group. Whether the use of the metabolites of the glycolysis, in addition to established biomarkers, could improve diagnostic accuracy requires further research.

## Data Availability

The datasets generated for this study are available on request to the corresponding author.

## Ethics Statement

This study was carried out in accordance with the recommendations of the ethical committee of the Martin-Luther university Halle-Wittenberg with written informed consent from all subjects. All subjects gave written informed consent in accordance with the Declaration of Helsinki. The protocol was approved by the ethical committee of the Martin-Luther-University Halle-Wittenberg (medical faculty).

## Author Contributions

ANS designed and analyzed the data, and wrote the manuscript. NB did the experimental part of the LC-MS measurements, analyzed the data, and wrote the manuscript. SM collected the patient’s samples and data and wrote the manuscript. DR and AS discussed the data and wrote the manuscript.

## Conflict of Interest Statement

The authors declare that the research was conducted in the absence of any commercial or financial relationships that could be construed as a potential conflict of interest.
