# Supplementary material for: Reduction of Glycolysis Intermediate Concentrations in the Cerebrospinal Fluid of Alzheimer’s Disease Patients
Source: Front Neurosci. 2019 Aug 21;13:871. doi: 10.3389/fnins.2019.00871 (PMC6713159; doi:10.3389/fnins.2019.00871)

Supplementary table 1: List of the metabolites analyzed by LC-MS in the CSF of AD and non-AD patients. *These metabolites were analyzed as one. d: detected: n.d: not detected.

| **Name** | **Abbreviation** | **Q1 m/z** | **Q3 m/z** | **CE in V** | **CXP in V** | **DP in V** | **Detection** |
| --- | --- | --- | --- | --- | --- | --- | --- |
| Pyruvate | PYR | 87.1 | 43 | -12 | -1 | -30 | **d** |
| Succinate | SUC | 117.1 | 73 | -10 | -3 | -35 | **d** |
| Oxaloacetate | OXA | 131.1 | 87 | -10 | -5 | -25 | **d** |
| Alpha-Ketoglutarate | AKG | 145.1 | 100.9 | -12 | -5 | -30 | **d** |
| Phosphoenol pyruvate | PEP | 167 | 78.8 | -20 | -5 | -35 | **d** |
| Dihydroxy acetone phosphate | DHAP | 169.1 | 97 | -14 | -7 | -35 | **d** |
| Aconitate | ACT | 173.1 | 84.9 | -18 | -5 | -35 | **d** |
| 2-Phosphoglycerate | 2PG | 185.1 | 79 | -44 | -3 | -35 | **d** |
| 3-Phosphoglycerate | 3PG | 185.1 | 79 | -44 | -3 | -35 | **d** |
| Ribulose-5-phosphate and Xylulose-5-phosphate | RIBU5P + XU5P | 229.1 | 96.8 | -16 | -5 | -45 | **d** |
| Glucose-6-phosphate* | G6P | 259.1 | 97 | -24 | -5 | -40 | **d** |
| Fructose-6-phosphate* | F6P | 259.1 | 96.9 | -20 | -5 | -40 | **d** |
| cyclic Adenosine monophosphate | cAMP | 328.2 | 133.9 | -36 | -5 | -125 | **d** |
| cyclic Guanosine monophosphate | cGMP | 344.2 | 150 | -34 | -11 | -70 | **d** |
| Adenosine monophosphate | AMP | 346.2 | 78.8 | -52 | -3 | -70 | **d** |
| Glutathione oxidized | GSSG | 611.6 | 306.1 | -34 | -7 | -35 | **d** |
| Glycine | GLY | 74.1 | 74 | -13 | -3 | -36 | **d** |
| Serine | SER | 104.1 | 74 | -16 | -3 | -20 | **d** |
| Proline | PRO | 114.1 | 86 | -18 | -3 | -55 | **d** |
| Threonine | THR | 118.1 | 73.9 | -18 | -3 | -25 | **d** |
| Leucine and Isoleucine | LEU + ILE | 261.3 | 130.2 | -10 | -1 | -30 | **d** |
| Asparagine | ASN | 131.1 | 87.1 | -16 | -11 | -75 | **d** |
| Aspartate | ASP | 132.1 | 88.1 | -18 | -13 | -50 | **d** |
| Glutamine | GLN | 145.1 | 108.9 | -18 | -5 | -30 | **d** |
| Lysine | LYS | 145.2 | 101 | -14 | -5 | -65 | **d** |
| Glutamate | GLA | 146.1 | 101.7 | -18 | -25 | -45 | **d** |
| Methionine | MET | 148.2 | 47 | -24 | -5 | -45 | **d** |
| Histidine | HIS | 154.2 | 93 | -24 | -3 | -40 | **d** |
| Orotate | ORO | 155.1 | 110.7 | -12 | -5 | -25 | **d** |
| Phenylalanine | PHE | 164.2 | 103 | -24 | -5 | -55 | **d** |
| Arginine | ARG | 173.2 | 131 | -18 | -7 | -50 | **d** |
| Tyrosine | TYR | 180.2 | 118.9 | -24 | -5 | -60 | **d** |
| Tryptophane | TRP | 203.2 | 116.2 | -22 | -7 | -50 | **d** |
| Cytidine | C | 242.2 | 108.9 | -18 | -5 | -70 | **d** |
| Uridine | U | 243.2 | 109.9 | -22 | -5 | -65 | **d** |
| 5-Aminoimidazole-4-carboxamide ribonucleotide | AICAR | 257.2 | 124.9 | -18 | -7 | -65 | **d** |
| Glucose-1-phosphate | G1P | 259.1 | 240.8 | -16 | -15 | -30 | **d** |
| Adenosine | A | 266.2 | 133.9 | -12 | -1 | -70 | **d** |
| Guanosine | G | 282.2 | 149.9 | -26 | -7 | -80 | **d** |
| Deoxyadenosine monophosphate | dAMP | 330.2 | 78.8 | -44 | -3 | -50 | **d** |
| Cytidine diphosphate | CDP | 402.2 | 78.9 | -70 | -5 | -65 | **d** |
| Uracil diphosphate glucose | UDP-glucose | 565.3 | 323 | -36 | -11 | -125 | **d** |
| Arginino succinate | ARGSUC | 289.3 | 271.3 | -14 | -15 | -55 | **d** |
| S-adenosyl homocysteine | SAH | 383.4 | 133.9 | -36 | -7 | -80 | **d** |
| Carbamoyl aspartic acid | CA | 175.1 | 131.8 | -16 | -7 | -25 | **d** |
| Carnithine | CARN | 220.2 | 145.9 | -12 | -7 | -35 | **d** |
| Citrulline | CITRUL | 174.2 | 131 | -18 | -7 | -35 | **d** |
| Creatine | CREA | 130.1 | 88.1 | -14 | -5 | -25 | **d** |
| Creatine phosphate | CREA-P | 210.1 | 78.9 | -22 | -1 | -35 | **d** |
| Farnesyl diphosphate | FARN-PP | 381.3 | 78.9 | -50 | -5 | -50 | **d** |
| Glyceol -3-phosphate | GLC3P | 171.1 | 78.8 | -24 | -1 | -45 | **d** |
| Lactate | LAC | 89.1 | 42.9 | -12 | -5 | -15 | **d** |
| S-Adenosyl methionine | SAM | 356 | 133.9 | -24 | -7 | -40 | **d** |
| Pantothenate | PAN | 218.2 | 88.1 | -18 | -5 | -55 | **d** |
| Glucose | glucose | 178.9 | 89 | -12 | -13 | -50 | **d** |
| Chloride | Cl- | 37 | 37 | -5 | -5 | -30 | **d** |
| Phosphate | PO4--- | 96.9 | 78.9 | -18 | -15 | -40 | **d** |
| Sulfate | SO4-- | 97 | 97 | -18 | -15 | -40 | **d** |
| Fumarate | FUM | 115.1 | 70.9 | -10 | -3 | -35 | **d** |
| Citrate | CIT | 191.1 | 87 | -26 | -5 | -35 | **d** |
| Isocitrate | ISOCIT | 191.1 | 73 | -28 | -3 | -40 | **d** |
| Bisphosphoglycerate | BPG | 265 | 167 | -20 | -1 | -40 | n.d |
| Malate | MAL | 133.1 | 115 | -14 | -9 | -30 | n.d |
| Glyceraldehyde-3-phosphate | GAP | 169.1 | 97 | -14 | -7 | -35 | n.d |
| Erythrose-4-phosphate | E4P | 199.1 | 96.8 | -16 | -5 | -45 | n.d |
| Ribose-5-phosphate | R5P | 229.1 | 96.8 | -20 | -5 | -35 | n.d |
| Sedoheptulose-7-phosphate | S7P | 289.2 | 97 | -22 | -5 | -50 | n.d |
| Gluthation reduced | GSH | 306.3 | 143 | -26 | -7 | -5 | n.d |
| Fructose-bisphosphate | FBP | 339.1 | 96.9 | -28 | -5 | -60 | n.d |
| Adenosine diphosphate | ADP | 426.2 | 78.9 | -66 | -3 | -75 | n.d |
| Nicotin amide adenine dinucleotide oxidized | NAD | 662.4 | 540.1 | -22 | -15 | -45 | n.d |
| Nicotin amide adenine dinucleotide reduced | NADH | 664.4 | 78.9 | -124 | -1 | -100 | n.d |
| Nicotine amide adenine dinucleotide phosphate oxidized | NADP | 742.4 | 620 | -22 | -17 | -55 | n.d |
| Nicotine amide adenine dinucleotide phosphate reduced | NADPH | 744.4 | 79 | -118 | -3 | -110 | n.d |
| Flavine adenine dinucleotide oxidized | FAD | 784.5 | 79 | -130 | -1 | -60 | n.d |
| Acetyl coenzyme A | AcCoA | 808.6 | 78.9 | -130 | -1 | -120 | n.d |
| Dihydro orotate | DHO | 157.1 | 112.7 | -10 | -5 | -40 | n.d |
| Alanine | ALA | 148 | 88.1 | -8 | -3 | -20 | n.d |
| Valine | VAL | 233.3 | 116 | -10 | -5 | -25 | n.d |
| Cystine | CYS | 239.3 | 120 | -32 | -1 | -40 | n.d |
| Ribose-1-phosphate | R1P | 229.1 | 211 | -14 | -3 | -50 | n.d |
| Deoxycytidine monophosphate | dCMP | 306.2 | 195 | -20 | -11 | -35 | n.d |
| Deoxyuracil monophosphate | dUMP | 307.2 | 194.9 | -22 | -11 | -40 | n.d |
| Cytidine monophosphate | CMP | 322.2 | 79 | -68 | -5 | -65 | n.d |
| Uridine monophosphate | UMP | 323.2 | 79 | -68 | -5 | -65 | n.d |
| 5-Aminoimidazole-4-carboxamide ribonucleotide phosphate | AICAR-P | 337.2 | 79.1 | -50 | -5 | -85 | n.d |
| Deoxyguanosine monophosphate | dGMP | 346.2 | 78.8 | -42 | -3 | -80 | n.d |
| Inosine monophosphate | IMP | 347.2 | 134.8 | -38 | -7 | -70 | n.d |
| Guanosine monophosphate | GMP | 362.2 | 78.9 | -66 | -5 | -65 | n.d |
| Xanthosine-5-phosphate | XAN5P | 363.2 | 151.1 | -36 | -5 | -60 | n.d |
| Cytidine diphosphate | dCDP | 386.2 | 78.9 | -56 | -3 | -70 | n.d |
| Phosphoribosyl diphosphate | PRPP | 389.1 | 176.8 | -28 | -9 | -55 | n.d |
| Deoxythymidine diphosphate | dTDP | 401.2 | 78.8 | -68 | -3 | -70 | n.d |
| Uridine diphosphate | UDP | 403.2 | 78.8 | -68 | -3 | -75 | n.d |
| Butyl coenzyme A | Bu-CoA | 417.8 | 78.8 | -74 | -3 | -50 | n.d |
| Acetoacetyl coenzyme A | AcetoAcCoA | 424.8 | 382.6 | -12 | -11 | -50 | n.d |
| Malonyl coenzyme A | Malo-CoA | 425.8 | 404.1 | -8 | -11 | -20 | n.d |
| Succinyl coenzyme A | Succ-CoA | 432.8 | 382.6 | -12 | -13 | -25 | n.d |
| Methylmalonyl coenzyme A | MM-CoA | 432.8 | 410.6 | -8 | -13 | -25 | n.d |
| Guanosine diphosphate | GDP | 442.2 | 78.9 | -70 | -3 | -85 | n.d |
| Hydroxy methylglutaryl coenzyme A | HMG-CoA | 454.8 | 382.6 | -18 | -9 | -50 | n.d |
| Adenylo succinate | Adenylo-Suc | 462.3 | 133.9 | -62 | -7 | -85 | n.d |
| Deoxycytidine triphosphate | dCTP | 466.2 | 158.7 | -44 | -13 | -75 | n.d |
| Deoxyuracil triphosphate | dUTP | 467.1 | 158.7 | -32 | -9 | -80 | n.d |
| Deoxythymidine triphosphate | dTTP | 481.2 | 158.7 | -38 | -9 | -80 | n.d |
| Cytidine triphosphate | CTP | 482.2 | 158.8 | -36 | -9 | -85 | n.d |
| Uracil triphosphate | UTP | 483.1 | 158.8 | -38 | -9 | -90 | n.d |
| Deoxyadenosine triphosphate | dATP | 490.2 | 158.7 | -36 | -11 | -75 | n.d |
| Guanosine triphosphate | GTP | 522.2 | 158.8 | -48 | -9 | -90 | n.d |
| Propionyl coenzyme A | Prop-CoA | 822.6 | 78.8 | -130 | -13 | -120 | n.d |
| Dephospho Coenzyme A | dpCoA | 686.6 | 78.8 | -112 | -1 | -105 | n.d |
| Isopentenyl diphosphate + Dimethylallyl diphosphate | IP-PP+DMA-PP | 245.1 | 78.8 | -30 | -3 | -50 | n.d |
| Glucosamine-1-phosphate | GA1P | 258.2 | 78.9 | -42 | -1 | -55 | n.d |
| Glucosamine-6-phosphate | GA6P | 258.2 | 97 | -24 | -5 | -45 | n.d |
| Geranyl diphosphate | GERA-PP | 313.2 | 78.9 | -46 | -1 | -65 | n.d |
| Ornithine | ORN | 131.2 | 82.9 | -20 | -5 | -60 | n.d |
| Mevalonate-5-phosphate | MEVA5P | 227.1 | 97 | -24 | -5 | -30 | n.d |
| Mevalonate | MEVA | 147.2 | 59.1 | -20 | -7 | -45 | n.d |
| Orotidine-5-phosphate | ORO5P | 390.2 | 78.9 | -78 | -1 | -50 | n.d |
| Adenosine triphosphate | ATP | 506 | 158.8 | -38 | -9 | -80 | n.d |
| Sucrose | sucrose | 341.1 | 89 | -38 | -13 | -240 | n.d |
| 6-Phosphogluconate | 6PG | 275 | 79 | -66 | -5 | -60 | n.d |

Supplementary table 2: Summary of the statistical analysis of the metabolites detected in the CSF of the AD and non-AD patients. In model 1 Least squares multiple regression analysis was used to analyze the relationship between the dependent variables (biomarker, represented by the area under the peak in relative units) and the independent variable (diagnosis). In the adjusted model age and gender were added as independent variables in the analyses. *These metabolites were analyzed as one. SE: Standard Error.

| Abbreviation |  |  | Model 1 unadjusted | | | |  | | Model 2 (adjusted for Age and Gender) | | | | | | |
| --- | --- | --- | --- | --- | --- | --- | --- | --- | --- | --- | --- | --- | --- | --- | --- |
|  | regression coefficient diagnosis | | SE |  | F-ratio | p-Value | regression coefficient | | | SE | | F-ratio | p-Value |  |  |
|  |  | |  |  |  |  | Age | Gender | | Age | Gender |  |  |  |  |
| PYR | -7.7x10^4^ | | 2.4x10^4^ |  | 10.0 | 2.6x10^-2^ | 1.1x10^3^ | 4.0x10^3^ | | 1.4x10^3^ | 2.8x10^4^ | 0.0 | 7.4x10^-1^ |  |  |
| SUC | -4.4x10^5^ | | 5.1x10^5^ |  | 1.0 | 3.9x10^-1^ | 1.0x10^4^ | -3.1x10^5^ | | 2.8x10^4^ | 5.5x10^5^ | 0.0 | 7.4x10^-1^ |  |  |
| OXA | 1.7x10^5^ | | 1.5x10^5^ |  | 1.0 | 2.6x10^-1^ | 6.4x10^3^ | -1.8x10^5^ | | 8.6x10^3^ | 1.6x10^5^ | 1.0 | 3.0x10^-1^ |  |  |
| AKG | -4.8x10^4^ | | 4.8x10^4^ |  | 1.0 | 3.2x10^-1^ | -1.3x10^3^ | -6.4x10^4^ | | 2.6x10^3^ | 5.1x10^4^ | 1.0 | 4.5x10^-1^ |  |  |
| PEP | -6.4x10^-4^ | | 1.3x10^-4^ |  | 21.0 | <1x10^4^ | -1.7x10^3^ | -1.9x10^4^ | | 8.6x10^2^ | 1.6x10^4^ | 2.0 | 1.0x10^-1^ |  |  |
| DHAP | -3.6x10^4^ | | 1.4x10^4^ |  | 6.0 | 1.7x10^-2^ | -1.5x10^2^ | -1.1x10^4^ | | 8.5x10^2^ | 1.6x10^4^ | 0.0 | 7.7x10^-1^ |  |  |
| ACT | -8.4x10^5^ | | 4.4x10^5^ |  | 4.0 | 6.0x10^-2^ | 3.5x10^4^ | -4.8x10^5^ | | 2.4x10^4^ | 4.7x10^5^ | 2.0 | 1.3x10^-1^ |  |  |
| 2PG | -1.2x10^4^ | | 3.9x10^3^ |  | 10.0 | 2.8x10^-3^ | -7.8x10^2^ | -7.6x10^3^ | | 2.0x10^2^ | 4.0x10^3^ | 7.0 | 1.4x10^-3^ |  |  |
| 3PG | -1.2x10^5^ | | 2.9x10^4^ |  | 19.0 | 1.0x10^-4^ | -2.7x10^3^ | -1.7x10^4^ | | 1.8x10^3^ | 3.5x10^4^ | 1.0 | 3.3x10^-1^ |  |  |
| RIBU5P + XU5P | 1.2x10^5^ | | 9.3x10^3^ |  | 3.0 | 9.1x10^-1^ | -5.2x10^2^ | 7.2x10^3^ | | 5.1x10^2^ | 1.0x10^4^ | 1.0 | 3.6x10^-1^ |  |  |
| G6P* | -1.1x10^4^ | | 1.2x10^4^ |  | 1.0 | 3.6x10^-1^ | 1.7x10^2^ | 1.0x10^4^ | | 6.8x10^2^ | 1.3x10^4^ | 0.0 | 7.1x10^-1^ |  |  |
| F6P* | * | | * |  | * | * | * | * | | * | * | * | * |  |  |
| cAMP | -1.1x10^3^ | | 3.4x10^3^ |  | 0.1 | 7.4x10^-1^ | -1.0x10^4^ | 2.7x10^3^ | | 1.9x10^2^ | 3.6x10^3^ | 0.3 | 7.3x10^-1^ |  |  |
| cGMP | -1.7x10^3^ | | 1.2x10^3^ |  | 1.8 | 1.8x10^-1^ | -3.6x10^1^ | -9.8x10^2^ | | 7.1x10^1^ | 1.3x10^3^ | 0.3 | 7.3x10^-1^ |  |  |
| AMP | 3.6x10^1^ | | 4.3x10^2^ |  | 0.0 | 9.3x10^-1^ | -5.3x10^1^ | 9.66x10^2^ | | 2.2x10^1^ | 4.33x10^2^ | 4.2 | 2.0x10^-2^ |  |  |
| GSSG | -8.0x10^2^ | | 5.1x10^2^ |  | 2.4 | 1.2x10^-1^ | -2.0x10^1^ | 6.5x10^2^ | | 2.8x10^1^ | 5.5x10^2^ | 1.2 | 2.9x10^-1^ |  |  |
| GLY | 3.1x10^3^ | | 2.6x10^3^ |  | 1.4 | 2.4x10^-1^ | 1.2x10^2^ | -3.3x10^3^ | | 1.4x10^2^ | 2.8x10^3^ | 1.5 | 2.3x10^-1^ |  |  |
| SER | 2.4x10^3^ | | 2.2x10^3^ |  | 1.2 | 2.8x10^-1^ | 8.9x10^1^ | -4.4x10^3^ | | 1.2x10^2^ | 2.3x10^3^ | 2.6 | 8.1x10^-2^ | |  |
| PRO | -6.8x10^2^ | | 6.2x10^2^ |  | 1.2 | 2.7x10^-1^ | 0.9x10^1^ | 4.8x10^2^ | | 3.4x10^1^ | 6.7x10^2^ | 0.2 | 7.6x10^-1^ | |  |
| THR | 3.1x10^3^ | | 8.4x10^3^ |  | 0.0 | 7.0x10^-1^ | 1.6x10^1^ | 2.3x10^3^ | | 1.2x10^2^ | 2.3x10^3^ | 0.5 | 6.1x10^-1^ | |  |
| LEU + ILE | -8.5x10^2^ | | 1.0x10^3^ |  | 0.6 | 4.1x10^-1^ | 4.0x10^1^ | -3.5x10^1^ | | 5.5x10^1^ | 1.1x10^3^ | 0.2 | 7.6x10^1^ | |  |
| ASN | -2.9x10^2^ | | 2.0x10^2^ |  | 2.1 | 1.4x10^-1^ | 0.3x10^1^ | 8.7x10^1^ | | 1.1x10^1^ | 2.2x10^2^ | 0.0 | 9.0x10^-1^ | |  |
| ASP | 1.9x10^3^ | | 4.9x10^3^ |  | 0.0 | 6.9x10^-1^ | 3.8x10^2^ | -1.6x10^3^ | | 2.6x10^2^ | 5.1x10^3^ | 1.0 | 2.8x10^-1^ | |  |
| GLN | 6.0x10^4^ | | 3.4x10^4^ |  | 3.0 | 8.0x10^-2^ | 2.3x10^3^ | 3.2x10^4^ | | 1.9x10^3^ | 3.7x10^4^ | 1.0 | 3.9x10^-1^ | |  |
| LYS | 5.4x10^3^ | | 4.0x10^3^ |  | 2.0 | 1.8x10^-1^ | 1.1x10^2^ | 4.0x10^3^ | | 2.2x10^2^ | 4.3x10^3^ | 0.0 | 6.2x10^-1^ | |  |
| GLA | -6.5x10^3^ | | 7.1x10^3^ |  | 1.0 | 3.5x10^-1^ | 8.8x10^1^ | -7.5x10^3^ | | 3.9x10^2^ | 7.5x10^3^ | 1.0 | 5.3x10^-1^ | |  |
| MET | 1.1x10^3^ | | 2.7x10^3^ |  | 0.2 | 6.7x10^-1^ | 2.1x10^2^ | -7.3x10^2^ | | 1.4x10^2^ | 2.8x10^3^ | 1.3 | 2.7x10^-1^ | |  |
| HIS | 1.3x10^3^ | | 1.4x10^3^ |  | 0.8 | 3.6x10^-1^ | 1.8x10^1^ | -1.5x10^-2^ | | 8.1x10^1^ | 1.5x10^3^ | 0.0 | 9.6x10^-1^ | |  |
| ORO | -1.2x10^4^ | | 1.1x10^4^ |  | 1.0 | 2.9x10^-1^ | 6.5x10^2^ | -5.8x10^3^ | | 6.2x10^2^ | 1.2x10^4^ | 1.0 | 4.3x10^-1^ | |  |
| PHE | 1.3x10^5^ | | 1.3x10^5^ |  | 1.0 | 3.3x10^-1^ | 1.5x10^4^ | -3.3x10^4^ | | 7.3x10^3^ | 1.4x10^5^ | 3.0 | 7.8x10^-2^ | |  |
| ARG | -1.1x10^4^ | | 6.0x10^3^ |  | 4.0 | 5.2x10^-2^ | -4.7x10^1^ | -8.2x10^3^ | | 3.3x10^2^ | 6.5x10^3^ | 1.0 | 4.4x10^-1^ | |  |
| TYR | -2.8x10^3^ | | 4.3x10^4^ |  | 0.0 | 9.4x10^-1^ | 2.3x10^3^ | -9.4x10^3^ | | 2.3x10^3^ | 4.5x10^4^ | 1.0 | 5.5x10^-1^ | |  |
| TRP | -1.9x10^2^ | | 2.2x10^3^ |  | 0.0 | 9.3x10^-1^ | 1.6x10^1^ | 2.3x10^3^ | | 1.2x10^2^ | 2.3x10^3^ | 0.5 | 6.1x10^-1^ | |  |
| C | 5.1x10^2^ | | 9.1x10^2^ |  | 0.3 | 5.7x10^-1^ | 4.9x10^5^ | 2.6x10^5^ | | 3.3x10^5^ | 6.4x10^6^ | 1.0 | 3.3x10^-1^ | |  |
| U | -3.2x10^4^ | | 2.9x10^4^ |  | 1.0 | 2.6x10^-1^ | -1.0x10^3^ | 1.7x10^3^ | | 1.6x10^3^ | 3.1x10^4^ | 0.0 | 7.9x10^-1^ | |  |
| AICAR | 2.0x10^3^ | | 2.1x10^3^ |  | 0.9 | 3.4x10^-1^ | 2.7x10^2^ | 1.3x10^3^ | | 1.1x10^2^ | 2.1x10^3^ | 2.9 | 6.6x10^-2^ | |  |
| G1P | -5.1x10^2^ | | 6.6x10^3^ |  | 0.0 | 9.3x10^-1^ | 5.3x10^2^ | 4.6x10^3^ | | 3.5x10^2^ | 6.9x10^3^ | 1.0 | 3.1x10^-1^ | |  |
| A | 1.0x10^2^ | | 2.0x10^2^ |  | 0.2 | 6.1x10^-1^ | 3.2x10^1^ | 1.3x10^2^ | | 1.0x10^1^ | 2.0x10^2^ | 4.7 | 1.2x10^-2^ | |  |
| G | 5.0x10^3^ | | 2.9x10^3^ |  | 2.9 | 9.0x10^-2^ | 2.4x10^2^ | -3.9x10^3^ | | 1.5x10^2^ | 3.0x10^3^ | 3.7 | 7.0x10^-2^ | |  |
| dAMP | -2.7x10^2^ | | 1.2x10^3^ |  | 0.0 | 8.3x10^-1^ | -6.0x10^1^ | -2.8x10^3^ | | 6.8x10^1^ | 1.3x10^3^ | 2.4 | 1.0x10^-1^ | |  |
| CDP | -2.9x10^2^ | | 1.4x10^2^ |  | 3.7 | 5.6x10^-2^ | -1.8x10^1^ | -2.8x10^2^ | | 0.8x10^1^ | 1.5x10^2^ | 3.3 | 5.1x10^-2^ | |  |
| UDP-glucose | -2.0x10^3^ | | 1.8x10^3^ |  | 1.3 | 2.6x10^-1^ | -0.2x10^1^ | 3.1x10^3^ | | 9.8x10^2^ | 1.8x10^3^ | 1.4 | 2.4x10^-1^ | |  |
| ARGSUC | -9.2x10^3^ | | 1.1x10^4^ |  | 1.0 | 4.0x10^-1^ | 1.2x10^3^ | 8.6x10^3^ | | 5.8x10^2^ | 1.1x10^4^ | 2.0 | 1.0x10^-1^ | |  |
| SAH | 2.7x10^3^ | | 1.6x10^3^ |  | 2.7 | 1.0x10^-1^ | 2.5x10^2^ | 1.7x10^3^ | | 8.7x10^1^ | 1.6x10^3^ | 4.2 | 2.7x10^-2^ | |  |
| CA | -1.8x10^3^ | | 9.5x10^2^ |  | 3.6 | 6.0x10^-2^ | -8.9x10^1^ | -1.5x10^3^ | | 5.3x10^1^ | 1.0x10^3^ | 2.0 | 1.4x10^-1^ | |  |
| CARN | -2.6x10^2^ | | 2.3x10^2^ |  | 1.2 | 2.6x10^-1^ | 1.1x10^1^ | 6.2x10^1^ | | 1.3x10^1^ | 2.5x10^2^ | 0.3 | 6.7x10^-1^ | |  |
| CITRUL | 1.0x10^3^ | | 3.1x10^3^ |  | 0.1 | 7.4x10^-1^ | 2.4x10^2^ | 1.1x10^3^ | | 1.6x10^2^ | 3.2x10^3^ | 1.0 | 3.6x10^-1^ | |  |
| CREA | -3.8x10^3^ | | 8.7x10^3^ |  | 0.0 | 6.5x10^-1^ | -2.1x10^2^ | -2.1x10^4^ | | 4.5x10^2^ | 8.7x10^3^ | 3.0 | 5.1x10^-2^ | |  |
| CREA-P | -8.5x10^3^ | | 2.8x10^4^ |  | 0.1 | 7.7x10^-1^ | -9.3x10^2^ | -5.9x10^4^ | | 1.5x10^3^ | 2.9x10^4^ | 2.0 | 1.4x10^-1^ | |  |
| FARN-PP | 6.4x10^2^ | | 3.9x10^2^ |  | 2.6 | 1.1x10^-1^ | 2.5x10^1^ | 3.7x10^2^ | | 2.1x10^1^ | 4.2x10^2^ | 0.8 | 4.2x10^-1^ | |  |
| GLC3P | -1.5x10^5^ | | 1.2x10^5^ |  | 2.0 | 2.1x10^-1^ | -1.1x10^3^ | -1.2x10^5^ | | 6.8x10^3^ | 1.3x10^5^ | 0.0 | 6.5x10^-1^ | |  |
| LAC | -5.2x10^4^ | | 1.1x10^5^ |  | 0.0 | 7.0x10^-1^ | 8.5x10^3^ | -1.4x10^5^ | | 7.5x10^3^ | 1.4x10^5^ | 2.0 | 2.2x10^-1^ | |  |
| SAM | 2.5x10^2^ | | 1.2x10^2^ |  | 3.9 | 5.1x10^-2^ | 1.1x10^1^ | 2.3x10^2^ | | 0.7x10^1^ | 1.3x10^2^ | 2.1 | 1.2x10^-1^ | |  |
| PAN | -2.2x10^4^ | | 2.5x10^5^ |  | 1.0 | 3.8x10^-1^ | 6.1x10^3^ | -3.7x10^5^ | | 1.3x10^4^ | 2.6x10^5^ | 1.0 | 2.6x10^-1^ | |  |
| glucose | -4.4x10^4^ | | 2.1x10^4^ |  | 4.0 | 4.4x10^-2^ | -8.5x10^2^ | -2.3x10^3^ | | 1.2x10^3^ | 2.4x10^4^ | 0.0 | 7.8x10^-1^ | |  |
| Cl- | 4.3x10^4^ | | 3.1x10^4^ |  | 2.0 | 1.7x10^-1^ | -8.9x10^2^ | -1.2x10^4^ | | 1.7x10^3^ | 3.4x10^4^ | 0.0 | 8.5x10^-1^ | |  |
| PO4--- | 1.4x10^6^ | | 1.2x10^6^ |  | 1.0 | 2.8x10^-1^ | 7.7x10^4^ | -4,2x10^5^ | | 7.1x10^4^ | 1.3x10^6^ | 1.0 | 4.6x10^-1^ | |  |
| SO4-- | 8.9x10^7^ | | 4.4x10^6^ |  | 4.0 | 5.1x10^-2^ | 6.1x10^5^ | 2.2x10^6^ | | 2.4x10^5^ | 4.6x10^6^ | 3.0 | 5.1x10^-2^ | |  |
| FUM | -1.4x10^3^ | | 6.9x10^3^ |  | 0.0 | 8.3x10^-1^ | 5.6x10^2^ | 5.0x10^3^ | | 3.7x10^2^ | 7.1x10^3^ | 1.0 | 3.0x10^-1^ | |  |
| CIT | 3.0x10^5^ | | 6.2x10^6^ |  | 0.0 | 9.6x10^-1^ | 4.9x10^5^ | 2.6x10^5^ | | 3.3x10^5^ | 6.4x10^6^ | 1.0 | 3.3x10^-1^ | |  |
| ISOCIT | -1.4x10^5^ | | 2.0x10^5^ |  | 1.0 | 4.6x10^-1^ | 2.3x10^4^ | 4.4x10^4^ | | 1.0x10^4^ | 2.0x10^5^ | 2.0 | 9.7x10^-2^ | |  |

Supplementary table 3: Statistical analysis for the metabolites of the glycolysis. Least squares multiple regression analysis was used to analyze the relationship between the dependent variable (metabolite) and the independent variables (age, gender, disease). N: number of samples analyzed (22 AD, 33 non-AD); SE: standard error. A p < 0.05 was considered statistically significant (in grey).

| Metabolite | N | Regression coefficient | | | SE | | | Independent variables p-Value | | | F-ratio | Significance level p-Value |
| --- | --- | --- | --- | --- | --- | --- | --- | --- | --- | --- | --- | --- |
|  |  | Age | Gender | Diagnosis | Age | Gender | Diagnosis | Age | Gender | Diagnosis |  |  |
| DHAP | 55 | 4.4x10^-4^ | -1.1x10^2^ | -2.5x10^-2^ | 4.3x10^-4^ | 7.8x10^-3^ | 8.3x10^-3^ | 3.1x10^-1^ | 1.5x10^-1^ | 3.8x10^-3^ | 3.2 | 2.8x10^-2^ |
| Glucose | 55 | 2.2x10^0^ | -1.7x10^2^ | -7.1x10^2^ | 1.9x10^1^ | 3.6x10^2^ | 3.8x10^2^ | 9.0x10^-1^ | 6.2x10^-1^ | 6.9x10^-2^ | 1.3 | 2.7x10^-1^ |
| G6P + F6P | 55 | 4.7x10^-4^ | 8.9x10^-3^ | -1.3x10^-2^ | 8.2x10^-4^ | 1.4x10^-2^ | 1.5x10^-2^ | 5.6x10-^1^ | 5.5x10-^1^ | 3.8x10-^1^ | 0.4 | 6.9x10^-1^ |
| 2PG | 55 | -5.7x10^-4^ | -1.0x10^2^ | -1.0x10^-2^ | 2.2x10^-4^ | 4.0x10^-3^ | 4.2x10^-3^ | 1.2x10^-2^ | 1.5x10^-2^ | 1.3x10^-2^ | 7.7 | 2.0x10^-4^ |
| 3PG | 55 | 2.1x10^-4^ | -4.8x10^-2^ | -1.4x10^-1^ | 1.7x10^-3^ | 3.2x10^-2^ | 3.3x10^-2^ | 9.0x10^-1^ | 1.3x10^-1^ | 1.0x10^-4^ | 7.1 | 4.0x10^-4^ |
| PEP | 55 | -5.1x10^-4^ | -5.7x10^-2^ | -1.1x10^-1^ | 1.3x10^-3^ | 2.4x10^-2^ | 2.5x10^-2^ | 6.9x10^-1^ | 2.0x10^-2^ | <1.0x10^-4^ | 9.5 | <1.0x10^-4^ |
| PYR | 55 | 6.7x10^-1^ | -5.3x10^0^ | -1.6x10^2^ | 3.0x10^-1^ | 5.4x10^0^ | 5.8x10^0^ | 2.8x10^-2^ | 3.3x10^-1^ | 5.2x10^-3^ | 3.4 | 2.2x10^-2^ |

Supplementary Figure 1: Standard curves for all glycolysis intermediates with significant changes between the analyzed patient groups. Standard curves of the quantified metabolites show high linearity in the concentration ranges detected in the CSF samples. Note that 2PG was also used for the quantification of its isomers 3PG.


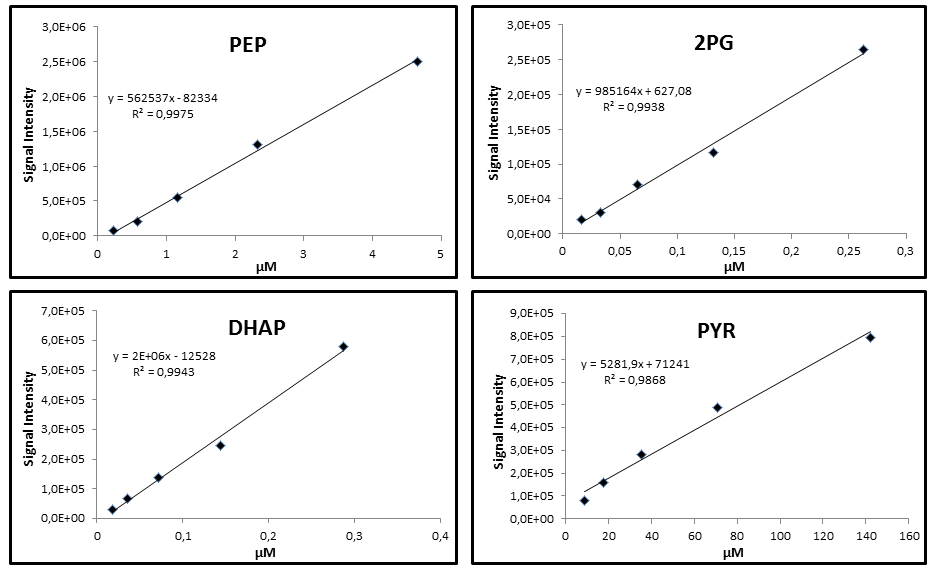


Supplementary Figure 2: Correlation of the Aβ1-42/Aβ1-40 ratio with the metabolites of the glycolysis.


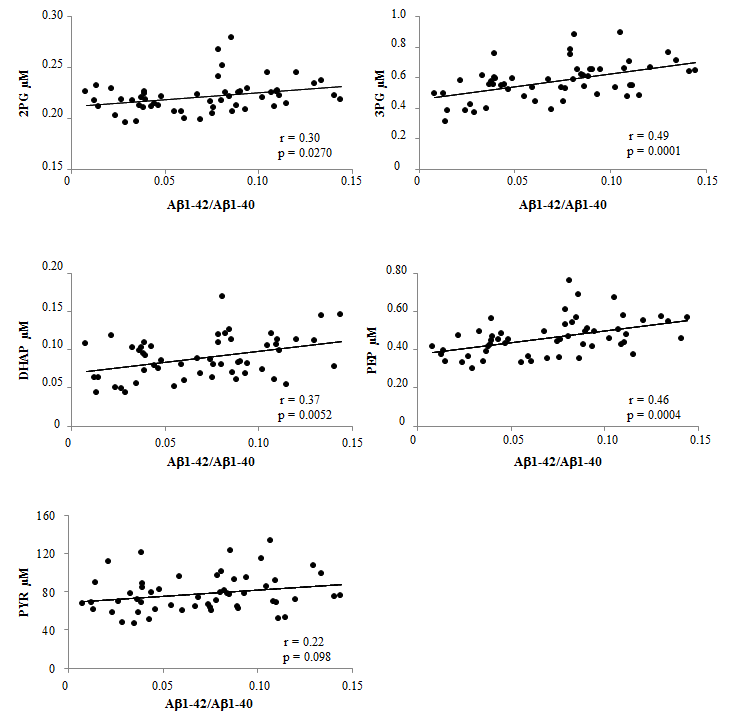

Supplement: Supplementary file 1 [file Data_Sheet_1.docx]
